# Antinociceptive actions of honokiol and magnolol on glutamatergic and inflammatory pain

**DOI:** 10.1186/1423-0127-16-94

**Published:** 2009-10-16

**Authors:** Yi-Ruu Lin, Hwei-Hsien Chen, Yu-Chin Lin, Chien-Hsin Ko, Ming-Huan Chan

**Affiliations:** 1 Institute of Pharmacology and Toxicology, Tzu Chi University, Hualien, Taiwan; 2 Department of Biotechnology, Transworld Institute of Technology, Yunlin, Taiwan; 3 Department of Chinese Medicine, Buddhist Tzu Chi General Hospital, Hualien, Taiwan

## Abstract

The antinociceptive effects of honokiol and magnolol, two major bioactive constituents of the bark of *Magnolia officinalis*, were investigated on animal paw licking responses and thermal hyperalgesia induced by glutamate receptor agonists including glutamate, N-methyl-D-aspartate (NMDA), and metabotropic glutamate 5 receptor (mGluR5) activator (RS)-2-chloro-5-hydroxyphenylglycine (CHPG), as well as inflammatory mediators such as substance P and prostaglandin E_2 _(PGE_2_) in mice. The actions of honokiol and magnolol on glutamate-induced c-Fos expression in the spinal cord dorsal horn were also examined. Our data showed that honokiol and magnolol blocked glutamate-, substance P- and PGE_2_-induced inflammatory pain with similar potency and efficacy. Consistently, honokiol and magnolol significantly decreased glutamate-induced c-Fos protein expression in superficial (I-II) laminae of the L4-L5 lumbar dorsal horn. However, honokiol was more selective than magnolol for inhibition of NMDA-induced licking behavioral and thermal hyperalgesia. In contrast, magnolol was more potent to block CHPG-mediated thermal hyperalgesia. These results demonstrate that honokiol and magnolol effectively decreased the inflammatory pain. Furthermore, their different potency on inhibition of nociception provoked by NMDA receptor and mGluR5 activation should be considered.

## Background

In inflammatory pain, the neurotransmitters (glutamate and serotonin), neuromodulators (substance P) and inflammatory mediators (bradykinin, histamine and prostaglandin) released from the primary afferents and dorsal horn neurons under tissue damage play an important role in eliciting the hyperactivity on nociceptive behaviors [1-5]. A number of evidence has further supported the development of central hyperexcitability and persistent pain relating to the activation of N-methyl-D-aspartate (NMDA) receptors [6]. The glutamatergic system is well known to involve in the inflammatory hyperalgesia, since NMDA and non-NMDA receptor antagonists attenuate the nociceptive behaviors which are associated with inflammatory responses in various animal models [7]. Recently, we have observed that honokiol and magnolol, isolated from the bark of *Magnolia officinals *[8,9], possess the ability to block the glutamate receptor-mediated intracellular cation signals [10] and inhibit glutamate-induced cell damage [11]. According to the above evidence, it is proposed that honokiol and magnolol may have the potent analgesic activity via blockade of glutamate receptors.

Magnolol has been reported to inhibit acetic acid-induced nociceptive response, and to reduce the hind-paw edema induced by carrageenan, compound 48/80, and polymyxin B in mice [12]. Our previous study also demonstrates that honokiol and magnolol block the inflammatory phase of the overt nociception induced by formalin. However, they do not produce analgesia in tail-flick, hot-plate paw-licking and the neurogenic phase of formalin-induced licking response [13]. Thus, we suggest that honokiol and magnolol may not affect the thermal pain, but reduce the inflammatory pain. Therefore, the antinociceptive mechanisms of honokiol and magnolol particularly in inflammatory pain require further investigation.

In the present study, we evaluated the antinociceptive actions of honokiol and magnolol on pain behaviors induced by glutamate receptor agonists including glutamate, NMDA, and mGluR5 agonist (RS)-2-chloro-5-hydroxyphenylglycine (CHPG), and inflammatory mediators such as substance P and prostaglandin E_2 _(PGE_2_), and also characterized their effects on glutamate-induced c-Fos expression in the spinal cord. Our results showed that honokiol and magnolol produced antinociceptive effects against glutamate-, NMDA-, CHPG-, substance P- and PGE_2_-induced inflammatory pain. Honokiol was more selective for inhibition of NMDA-induced licking behavioral and thermal hyperalgesia. Alternatively, magnolol was more potentially to block CHPG-mediated thermal hyperalgesia. Furthermore, honokiol and magolol also significantly decreased c-Fos expression induced by glutamate in the spinal cord.

## Materials and methods

### Animals

Male NMRI mice (8-9 weeks, 33-39 g) were supplied from the Laboratory Animal Center of Tzu Chi University (Hualien, Taiwan) and housed 4-5 animals per cage in a 12 hr light/dark cycle with free access to food and water. The experimental protocol was approved by Review Committee of the Tzu Chi University for the use of animal.

### Glutamate-induced nociceptive test

Investigation of excitatory amino acid (EAA), glutamate, involving in nociception has been described previously [14]. Briefly, a volume of 20 μl of glutamate (300 μg) was intraplantarly (i.pl.) injected into the ventral surface of the hind paw. Animals were observed individually for 30 min following glutamate injection. The amount of time spent licking the injected paw was recorded with a chronometer and was considered as indicative of nociception. For examining the antinociceptive effects, honokiol and magnolol (5 and 10 mg/kg, i.p.) were applied to animals 20 min before glutamate injection (i.pl.). Control animals received a similar volume of vehicle (corn oil, 10 ml/kg).

### NMDA- and PGE_2_-induced nociceptive test

NMDA (150 μg) or PGE_2 _(0.5 μg) were intraplantarly (i.pl.) injected into the ventral surface of the hind paw. Animals were observed individually for 5 min following NMDA or PGE_2 _injection. The amount of time spent licking the injected paw was recorded with a chronometer and was considered as indicative of nociception. Animals were pretreated with vehicle, honokiol and magnolol (0.1 to 1.0 μg/paw, i.pl.) 15 min before NMDA or PGE_2 _injection.

### Measurement of thermal hyperalgesia

Thermal hypersensitivity was determined by animal hind paw withdrawal response in hot water (47.0 ± 0.1°C). In order to calm down the animal's anxiety, each mouse was wrapped in a sock before any testing. Then the hind paw was immersed into a water bath (47.0 ± 0.1°C) to measure the hind paw withdrawal latency. The initial paw-withdrawal latency (T_0_) was measured before any nociceptive chemical treatment and the cut-off time was limited to 10 sec in order to minimize tissue damage. The nociceptive chemicals such as glutamate, NMDA, CHPG, PGE_2 _or substance P were given as an intraplantar injection into one hind paw, which was then immersed into hot water for the observation of paw withdrawal latency (T_1_) at 5, 15, 30, 60 and 120 min after chemical injection. For a measurement of antinociceptive properties, honokiol and magnolol were intraperitoneally (5 or 10 mg/kg) injected 20 min before glutamate injection, or intraplantarly (0.05 to 1.0 μg/paw) treated 15 min before NMDA, CHPG, PGE_2 _or substance P injection. Control animals received an injection of an appropriate volume of vehicle. Data were expressed as a mean value of Δ paw-withdrawal latency which was calculated as T_0 _- T_1_.

### c-Fos immunohistochemistry

Mice were pretreated with vehicle (corn oil), honokiol or magnolol (10 mg/kg, i.p.) 20 minutes before injection of saline or glutamate into the plantar surface. Ninety minutes after injection of saline or glutamate, mice were deeply anaesthetized with thiopental (50 mg/kg, i.p.), then perfused transcardially with heparin solution (1000 USP/ml in 0.1 M PBS), and subsequently perfused with 100 ml of cold fixative (4% paraformaldehyde in 0.1 M PBS). The L4-L5 segments of spinal cord were removed, postfixed, and cryoprotected with 30% sucrose in 0.1 M PBS. Spinal cord tissues were then frozen and cut with a cryostat in frontal sections (50 μm thickness) which were collected in 0.1 M PBS for immunohistochemistry as described previously [15]. Rabbit primary antibody (Ab) for c-Fos (Santa Cruz, 1:5000) was used to identify the number of c-Fos labeled neurons of laminas I-II in randomly selected sections (4-5 sections per mouse).

c-Fos immunoreactivity was observed in the cell nuclei and shown as a brownish round dot in the spinal cord sections under a light microscope. Quantification of Fos positive nuclei were counted with the aid of a Nikon Eclipse 800 microscope, equipped with a Polaroid DMC digital camera (1600 × 1200 dpi in 8 bits) with 100× magnification using the Image Pro Plus 4.5 morphometry program (Media Cybernetics, Silver, MD). Following background subtraction, the threshold was adjusted so that pale- and deep-stained nuclei could be equally recognized by the counting program. Cell counts were made with the help of the Image Pro Plus 4.5 software and manually counted by an observer who was blind to the group assignment. The number of c-Fos-stained nuclei was counted in four sections per animal in superficial dorsal horn (laminae I and II) of the L4-L5 lumbar dorsal horn ipsilateral to the formalin-injected site.

### Materials

Honokiol and magnolol were purchased from Nacalai Tesque (Kyoto, Japan) and dissolved in corn oil as a stock solution. c-Fos antibody was purchased from Santa Cruz Biotechnology (Delaware Avenue, CA, USA). CHPG, metabotropic glutamate 5 receptor (mGluR5) agonist, was purchased from Tocris (Northpoint Fourth Way Avonmouth, UK). Other chemicals were obtained from Sigma (St. Louis, MO, USA).

### Data analysis

Most data are expressed as means ± S.E.M. Statistical significance of difference between groups was determined by two way repeated measures ANOVA, two-way ANOVA or one-way ANOVA followed by a Student-Newman-Keuls post-hoc test. A *P *value of less than 0.05 was considered statistically significant.

## Results

### Effects of honokiol and magnolol on nociceptive responses and thermal hyperalgesia induced by glutamate receptor agonists

**Figure 1:**
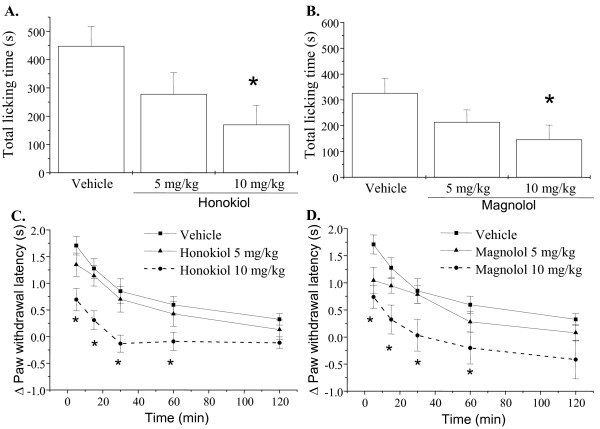
**Effects of honokiol and magnolol on glutamate-induced nociceptive responses and thermal hyperalgesia in mice**. Honokiol (5 and 10 mg/kg), magnolol (5 and 10 mg/kg) and vehicle (corn oil) were intraperitoneally administered 20 min prior to glutamate (300 μg) injection into hindpaw, and the total time spent licking the hindpaw was measured for 30 min after intraplantar injection of glutamate (A, B). The paw withdrawal latency in hot water at 47°C was measured during 5-120 min after intraplantar injection of glutamate (C, D). Each point represents the mean ± S.E.M. (n = 6-8). * indicates significantly different from vehicle group, *p < 0.05.

**Figure 2:**
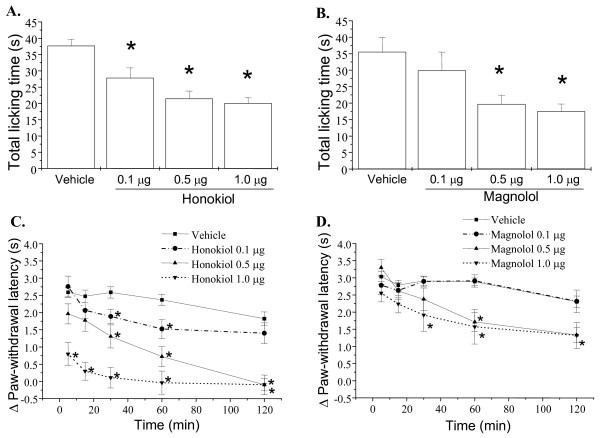
**Effects of honokiol and magnolol on NMDA-induced nociceptive responses and thermal hyperalgesia in mice**. Honokiol (0.1-1.0 μg/paw), magnolol (0.1-1.0 μg/paw) and vehicle were intraplantarly administered 20 min prior to NMDA (150 μg) injection into hindpaw, and the total time spent licking the hindpaw was measured for 5 min after intraplantar injection of NMDA (A, B). The paw withdrawal latency in hot water at 47°C was measured during 5-120 min after intraplantar injection of NMDA (C, D). Each point represents the mean ± S.E.M. (n = 8-16). * indicates significantly different from vehicle group, *p < 0.05.

To test whether the antinociceptive actions of honokiol and magnolol are involved in glutamatergic regulation, we assessed the effects of honokiol and magnolol on glutamate and NMDA induced licking response and thermal hyperalgesia in mice. As shown in Figure 1, honokiol and magnolol applied by intraperitoneal injection (i.p.) at 10 mg/kg significantly inhibited the glutamate-induced cumulative licking time and thermal hyperalgesia. Moreover, intraplantar application (i.pl.) of honokiol and magnolol (0.1-1.0 μg/paw) also concentration-dependently reduced the total licking time and thermal hyperalgesia induced by NMDA [(F(3,144) = 27.17, P < 0.001) and F(3,136) = 3.875, P = 0.017, respectively] (Fig. 2). However, magnolol at lower dose (0.1 μg/paw) did not significantly alter NMDA-induced nociceptive responses and thermal hyperalgesia (Fig. 2B and 2D).

**Figure 3:**
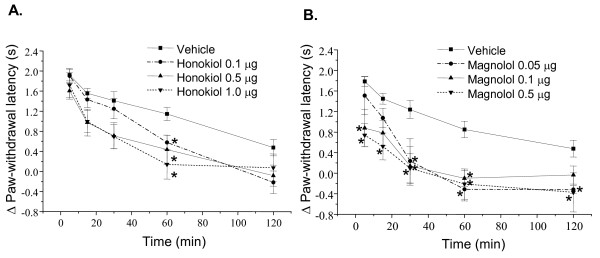
**Effects of honokiol and magnolol on CHPG-induced thermal hyperalgesia in mice**. Honokiol (0.1-1.0 μg/paw), magnolol (0.05-0.5 μg/paw) and vehicle were administered 20 min prior to CHPG (1 μg) injection into hindpaw. The paw withdrawal latency in the 47°C water was measured during 5-120 min after intraplantar injection of CHPG. Each point represents the mean ± S.E.M. (n = 7-19). * indicates significantly different from vehicle group, *p < 0.05.

The following study was undertaken to investigate whether the analgesia elicited by honokiol and magnolol is associated with the inhibition of metabotropic glutamate receptor (mGluR) activity. CHPG (1 μg/paw), a selective mGlu5 receptor agonist, was observed to induce a significant increase in the latency of thermal hyperalgesia. However, the thermal hyperalgesic effects of CHPG were inhibited by honokiol (0.1-1 μg/paw) and magnolol (0.05-0.5 μg/paw). Importantly, magnolol at lower does (0.05 μg/paw) could reduce CHPG-induced thermal hyperalgisia (Fig. 3).

### Effects of honokiol and magnolol on inflammatory mediator-induced nociceptive responses and thermal hyperalgesia

**Figure 4:**
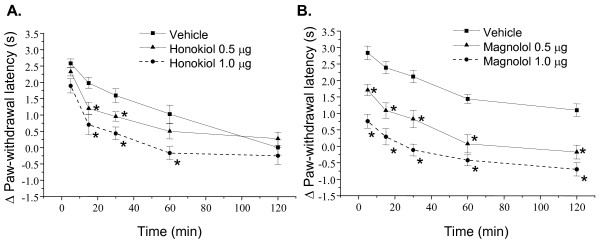
**Effects of honokiol and magnolol on substance P-induced thermal hyperalgesia in mice**. Honokiol (0.5 or 1.0 μg/paw), magnolol (0.5 or 1.0 μg/paw) and vehicle were administered 20 min prior to substance-P (0.5 μg) injection into hindpaw. The paw withdrawal latency in the 47°C water was measured during 5-120 min after intraplantar injection of substance P. Each point represents the mean ± S.E.M. (n = 7-9). * indicates significantly different from vehicle group, *p < 0.05.

**Figure 5:**
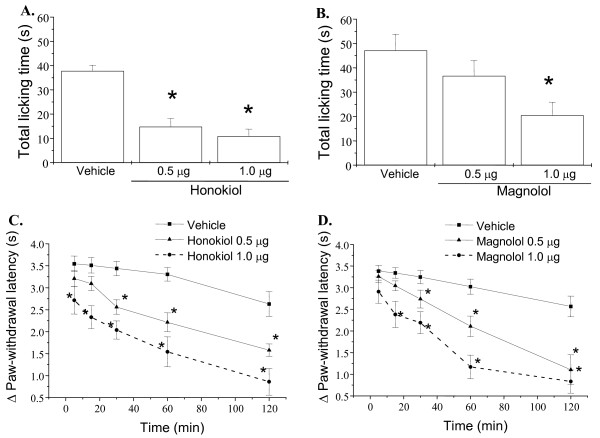
**Effects of honokiol and magnolol on PGE_2_-induced nociceptive responses and thermal hyperalgesia in mice**. Honokiol (0.5 or 1.0 μg/paw), magnolol (0.5 or 1.0 μg/paw) and vehicle were administered 20 min prior to PGE_2 _(0.5 μg) injection into hindpaw, and the total time spent licking the hindpaw was measured for 5 min after intraplantar injection of PGE_2 _(A, B). The paw withdrawal latency in the 47°C water was measured during 5-120 min after intraplantar injection of PGE_2 _(C, D). Each point represents the mean ± S.E.M. (n = 7-8). * indicates significantly different from vehicle group, *p < 0.05.

The intraplantar injection of substance P (0.5 μg/paw) and PGE_2 _(0.5 μg/paw) significantly decreased the paw withdrawal latency, which was gradually disappeared 2 h after injection (Fig. 4 and Fig. 5C, 5D). The thermal hyperalgesia induced by substance P was dose-dependently inhibited by honokiol (0.5-1.0 μg/paw, i.pl.) and magnolol (0.5-1.0 μg/paw, i.pl.) [F(2, 109) = 9.04, P = 0.002 and F(2, 92) = 42.5, P < 0.001, respectively] (Fig. 4).

Furthermore, honokiol (0.5-1.0 μg/paw, i.pl.) and magnolol (0.5-1.0 μg/paw, i.pl.) inhibited the nociceptive behaviors and thermal hyperalgesia [F(2, 84) = 1.182, P < 0.001 and F(2, 84) = 19.6, P < 0.001, respectively] induced by intraplantar injection of PGE_2 _(Fig. 5).

### Effect of honokiol and magnolol on glutamate-induced c-Fos expression

**Figure 6:**
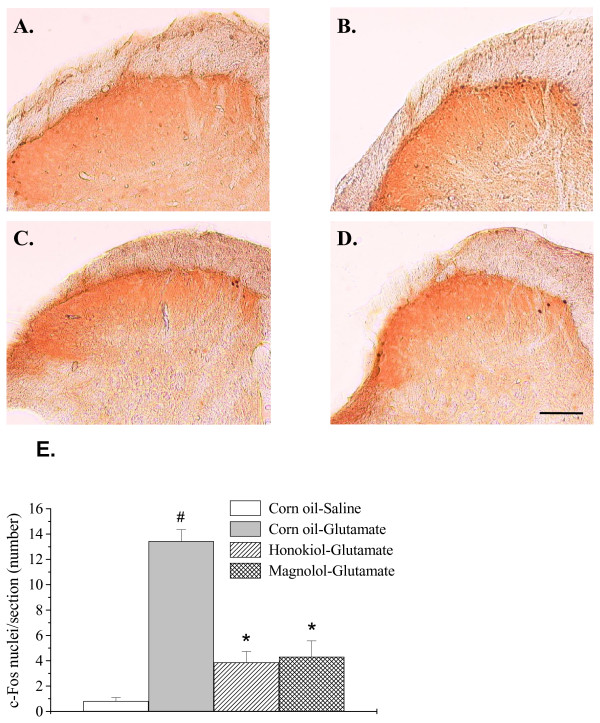
**Effects of honokiol and magnolol on glutamate-induced c-Fos protein expression in the spinal dorsal horn of the side ipsilateral to the injection**. Photomicrographs illustrating the four experimental groups are represented: corn oil i.p.+saline i.pl. (A), corn oil i.p.+glutamate i.pl. (B), honokiol (10 mg/kg) i.p.+glutamate i.pl. (C), and magnolol (10 mg/kg) i.p.+glutamate i.pl. (D). Summary data of glutamate-induced c-Fos protein expression after honokiol and magnolol treatment in superficial (I-II) spinal cord (E). Values are means ± S.E.M. (n = 4). # indicates significantly different from corn oil-saline group, #P < 0.05; * indicates significantly different from corn oil-glutamate group, *P < 0.05. Statistical analysis was determined by one-way ANOVA followed by a Student-Newman-Keuls post-hoc test. Scale bar = 100 μm.

The highest counts of c-Fos-positive cells were observed at superficial (I-II) laminae of the L4-L5 lumbar dorsal horn ipsilateral to the glutamate-injected site (Fig. 6B). In contrast, there were little or no c-Fos-positive cells in the contralateral spinal cord of glutamate injection or ipsilateral of saline-injected site (Fig. 6A). Honokiol (10 mg/kg) and magnolol (10 mg/kg) significantly decreased the glutamate-induced c-Fos protein expression in superficial spinal cord dorsal horn (Fig. 6).

## Discussion

Our previous report has indicated that honokiol and magnolol possess the ability to inhibit formalin-induced inflammatory nociception [13]. The present results further demonstrated that honokiol and magnolol attenuated glutamate-, NMDA-, mGluR5 agonist CHPG-, substance P- and PGE_2_-induced nociceptive responses. The inhibitory actions of honokiol on NMDA-induced licking behavioral and thermal hyperalgesia were more potent than magnolol. In contrast, magnolol had higher potency than honokiol to block CHPG-mediated thermal hyperalgesia. Importantly, both honokiol and magnolol significantly decreased glutamate-induced c-Fos protein expression in superficial (I-II) laminae of the L4-L5 lumbar dorsal horn. Taken together, these results indicated that the analgesic actions of honokiol and magnolol may be related to the blockade of glutamate receptor, NMDA receptor and mGlu5 receptor activation, and inflammation-mediated nociception.

Accumulating evidence has demonstrated that the excitatory amino acid glutamate and the glutamatergic receptors, both ionotropic and metabotropic glutamate receptors (iGluR and mGluRs), are critically contributed to nociceptive neurotransmission under the development and maintenance of pain responsiveness [16-19]. In response to noxious stimuli or tissue injury, excitatory amino acids are released in peripheral neurons or spinal cord [20,21]. Then activation of glutamate receptors, especially NMDA receptors, is involved in the development of spinal hyperexicitability and persistent pain transmission [6]. Consistently, NMDA receptor antagonists are reported to inhibit the spread of pain sensation and the hyperexcitability of spinal cord nociceptive neurons induced by C-fiber stimulation [22,23]. Furthermore, modulation of mGluRs can regulate peripheral nociceptor function. Importantly, activation of mGluR5 is contributed to the maintenance of peripheral nociceptive processes which are associated with inflammatory pain but not in physiological pain [24]. Peripheral injection of mGluR5 antagonist, 2-methyl-6-(phenylethynyl)-pyridine (MPEP), inhibits the inflammatory, but not the neurogenic phase of nociception induced by formalin [25]. However, activation of group III mGluRs, mGluR4 and mGluR7, just evokes the antinociceptive action on neuropathic pain [26]. In this regard, the inhibitory effects of honokiol and magonol on inflammatory pain mediated by mGluR5 activation were examined in this study. Here our results demonstrated that honokiol and magnolol reduced glutamate-, NMDA- and CHPG (mGluR5 agonist)-induced licking behavioral and thermal hyperalgesia, suggesting that the antinociceptive actions of honokiol and magnolol may be related to the inhibition of glutamate receptors including both NMDA and mGlu5 receptors.

It has been demonstrated that noxious stimuli-induced release of glutamate from nociceptive afferents in spinal cord, leading to the activation of either iGluR or mGluR on the postsynapse [21]. Fos, a nuclear phosphoprotein induced by a mammalian c-Fos protooncogene, is regarded as a maker of spinal activated neurons after noxious stimulation, such as thermal, mechanical and chemical stimuli [27]. Because c-Fos protein also contributes to behavioral hyperalgesia, the c-Fos expression in spinal cord after glutamate stimulation and the influences of honokiol and magnolol on glutamate-induced c-Fos expression were determined in this study. Here our data showed that honokiol and magnolol attenuated glutamate-induced c-Fos protein expression in superficial laminae of dorsal horn of the spinal cord. This inhibition on c-Fos expression was correlated to reduction in glutamate-induced nociceptive response and thermal hyperalgesia. The present finding is also in agreement with the inhibitory actions of NMDA and mGlu5 receptor antagonists on c-Fos expression in spinal neurons after noxious stimulation [28,29]. It is important to note that the antinociceptive potency of honokiol is higher than magnolol on NMDA-induced nociception, although their antinociceptive potency is similar to glutamate-induced nociception and c-Fos expression. These results are consistent to our previous observation for both compounds on blockade of glutamate- and NMDA-induced increases in [Ca^2+^]_i _or neurotoxicity in cerebellar granule cells [10,11]. In contrast, the antinociceptive potency of magnolol is more specific than honokiol on CHPG-induced thermal hyperalgesia. Therefore, our data further indicate that honokiol and magnolol may have the differential analgesic actions on NMDA and mGlu5 receptors. However, their influence on the activity of other subtypes of GluR is still unknown and required further study.

During an inflammatory event, nociception generation is a consequence of a complex interaction between a number of inflammatory mediators, including prostaglandins (PG), in inflamed tissues and the spinal cord. Notably, PGE_2 _is the prostanoids most relevant for the induction and maintenance of the inflammatory pain by either increasing the primary nociceptor responses or changing the spinal nociceptive signaling [30]. The nociceptive behavior and mechanical allodynia caused by PGE_2 _are attributed to the activation of G-protein-coupled EP receptors located in intrinsic spinal neurons (EP2) or primary afferent neurons (EP1, EP3, and EP3) and protein kinase dependent mechanisms [31,32]. Prostaglandins are also reported to enhance the release of glutamate and substance P from peripheral afferent fiber terminals, involving an increase in levels of cAMP and an increase in Ca^2+ ^and Na^+ ^conductance [33]. Furthermore, PGE_2_-induced hyperalgesia is abolished in NMDA receptor knock-out mice [34]. The present data showed that honokiol and magnolol not only inhibited the nociceptive response and thermal hyperlgesia induced by PGE_2 _but also blocked these responses induced by NMDA and glutamate. Thus, the blockade of glutamate receptors by honokiol and magnolol could contribute to their antinociception on PGE_2_-induced pain responses. However, the antinociceptive principles for interfering at the EP receptors or the following signaling transduction in PGE_2_-induced thermal hyperalgesia can not be excluded. Further study is required to figure out the analgesic mechanisms of honokiol and magnolol on PGE_2_-mediated nociceptive and inflammatory responses.

Upon noxious stimulation, tachykinins such as substance P and neurokinin A that are released from primary afferent fibers and excite spinal cord dorsal horn neurons bind to neurokinin receptors. It is believed that substance P induced signaling pathway is involved in the inflammatory process and in the transmission of sensory nociceptive information [30,35,36]. Although the release of substance P is facilitated by prostaglandins, substance P and glutamate conversely increase prostaglandin release [31]. It was observed that honokiol and magnolol significantly reduced substance P-mediated nociceptive signals, suggesting that the multiple antinociceptive actions of honokiol and magnolol may be via interaction with substance P-induced nociception and pain transmission in addition to prostaglandins and glutamate-mediated nociceptive responses. However, their pharmacological mechanisms still require corroboration in depth.

In conclusion, the present study demonstrates that honokiol and magnolol produce the antinociception against glutamate-, NMDA-, CHPG-, PGE_2_- and substance P-induced pain, and consistently decrease glutamate-induced c-Fos protein expression in superficial (I-II) laminae of the L4-L5 lumbar dorsal horn. It is noted that honokiol preferentially modulates the NMDA receptor-mediated nociception, whereas magnolol is more potent than honokiol to inhibit mGluR5-mediated response. Therefore, these results indicate that honokiol and magnolol may possess the therapeutic potential to treat the inflammatory pain and have the differential analgesic mechanisms on NMDA receptor and mGluR5 activation under nociceptive neurotransmission.

## Competing interests

The authors declare that they have no competing interests.

## Authors' contributions

YRL carried out the nociceptive tests and immunohistochemical assay, measured the thermal hyperalgesia, and drafted the manuscript. HHC participated in the design of the study and sequence alignment. YCL and CHK participated in the coordination and sequence alignment. MHC conceived of the study and helped to draft the manuscript. All authors read and approved the final manuscript.
